# Central Action of Peripherally Applied Botulinum Toxin Type A on Pain and Dural Protein Extravasation in Rat Model of Trigeminal Neuropathy

**DOI:** 10.1371/journal.pone.0029803

**Published:** 2012-01-04

**Authors:** Boris Filipović, Ivica Matak, Lidija Bach-Rojecky, Zdravko Lacković

**Affiliations:** 1 Laboratory of Molecular Neuropharmacology, Department of Pharmacology and Croatian Brain Research Institute, University of Zagreb School of Medicine, Zagreb, Croatia; 2 Department of Pharmacology, University of Zagreb School of Pharmacy and Biochemistry, Zagreb, Croatia; 3 Department of Otorhinolaryngology-Head and Neck Surgery, University Hospital Sveti Duh, Zagreb, Croatia; Southern Illinois University School of Medicine, United States of America

## Abstract

**Background:**

Infraorbital nerve constriction (IoNC) is an experimental model of trigeminal neuropathy. We investigated if IoNC is accompanied by dural extravasation and if botulinum toxin type A (BoNT/A) can reduce pain and dural extravasation in this model.

**Methodology/Principal Findings:**

Rats which developed mechanical allodynia 14 days after the IoNC were injected with BoNT/A (3.5 U/kg) into vibrissal pad. Allodynia was tested by von Frey filaments and dural extravasation was measured as colorimetric absorbance of Evans blue - plasma protein complexes. Presence of dural extravasation was also examined in orofacial formalin-induced pain. Unilateral IoNC, as well as formalin injection, produced bilateral dural extravasation. Single unilateral BoNT/A injection bilaterally reduced IoNC induced dural extravasation, as well as allodynia (lasting more than 2 weeks). Similarly, BoNT/A reduced formalin-induced pain and dural extravasation. Effects of BoNT/A on pain and dural extravasation in IoNC model were dependent on axonal transport through sensory neurons, as evidenced by colchicine injections (5 mM, 2 µl) into the trigeminal ganglion completely preventing BoNT/A effects.

**Conclusions/Significance:**

Two different types of pain, IoNC and formalin, are accompanied by dural extravasation. The lasting effect of a unilateral injection of BoNT/A in experimental animals suggests that BoNT/A might have a long-term beneficial effect in craniofacial pain associated with dural neurogenic inflammation. Bilateral effects of BoNT/A and dependence on retrograde axonal transport suggest a central site of its action.

## Introduction

In animals, long-lasting antinociception of peripherally applied botulinum toxin type A (BoNT/A) was reported in inflammatory pain induced by formalin [1], carrageenan and capsaicin [2], [3], peripheral neuropathic pain [4]–[8], postsurgical pain [9], experimental cystitis [10] and prostatitis [11]. Bilateral effect of unilaterally applied BoNT/A was demonstrated in different models [12]–[14]. Retrograde transport in sensory neurons and central site of BoNT/A action were suggested [12], [13] to explain this bilateral effect.

Infraorbital nerve constriction injury (IoNC) accompanied by hyperalgesia and allodynia is used as a model of trigeminal neuropathy in rats [15]. Standard antimigraine drugs, like 5-HT1B/1D receptor agonists and some antiepileptics reduce mechanical allodynia and hyperalgesia after the IoNC [16], [17]. BoNT/A pretreatment prevented the development of IoNC-induced trigeminal neuropathy [7], [8].

In the present study we found that: (1.) two types of pain in trigeminal region, IoNC and formalin injection, are, apart from mechanical allodynia, accompanied by dural protein extravasation; (2.) BoNT/A, besides bilateral reduction of pain, also bilaterally reduces dural extravasation in IoNC model; (3.) BoNT/A effects on pain and dural neurogenic inflammation are dependent on axonal transport in trigeminal sensory neurons.

## Materials and Methods

### Animals and Ethics statement

A total number of 200 male Wistar rats (300–350 g), bread in our animal facility (University of Zagreb School of Medicine, Croatia), were included in the study. Animals were kept under a constant 12 h/12 h light/dark cycle with unlimited access to food and water. The experiments were conducted according to the National Institute of Health Guide for the Care and Use of Laboratory Animals (Publication No. 85-23, revised 1996) and approved by the Ethical Committee of the University of Zagreb, School of Medicine (permit No. 07-76/2005-43).

### Drugs

The following drugs were used: botulinum toxin type A (Botox®, Allergan, Inc., Irvine, CA, USA); chloral hydrate, colchicine and formamide (Sigma, St. Louis, MO, USA); Evans blue (Merck KGaA, Darmstadt, Germany); formalin (Kemika, Zagreb, Croatia). Colchicine was reconstituted in 0.9% saline to obtain the 5 mM concentration. Each vial of Botox® contains 100 U of purified Clostridium botulinum type A neurotoxin complex. One unit of Botox® contains approximately 48 pg of whole molecular complex of BoNT/A, with molecular weight of 900 kDa. In order to obtain the dose needed, BoNT/A was reconstituted in adequate volume of 0.9% saline. Evans blue was reconstituted in 0.9% saline to obtain the required dose (40 mg/kg). Formalin was diluted to 2.5% concentration with saline.

BoNT/A was injected subcutaneously (s.c.) in a volume of 20 µl with a 27 ½ gauge syringe into the ipsilateral or contralateral vibrissal pad of conscious, restrained rats. Control animals were injected with saline. BoNT/A dose of 3.5 U/kg was chosen on the basis of previous experiments [1], [13], [18]. Colchicine (2 µl) was injected into the trigeminal ganglion (i.g.) of anesthetized rat as described by Neubert et al. [19]. Hamilton syringe needle (Hamilton Microliter #701, Hamilton, Bonaduz, Switzerland) was inserted through the skin into the infraorbital foramen, and advanced through the infraorbital canal and foramen rotundum into the trigeminal ganglion. Formalin was injected subcutaneously (s.c.) into the vibrissal pad of conscious, restrained rats in a volume of 50 µl with a 27 ½ gauge syringe.

### Surgery

Animals were anesthetized by a single intraperitoneal injection of chloral hydrate (300 mg/kg). Chronic constriction injury to the left infraorbital nerve was performed as described previously [15]. The skin on the left side of the forehead and nose of the rat was shaved. 1 ml of saline was injected into the medial portion of the orbit to elevate the eye in order to gain a better access to the infraorbital nerve. A mid-line scalp incision was made, exposing skull and nasal bone. Electrocauterization was used to prevent bleeding. The intraorbital part of infraorbital nerve was exposed using a modified surgical procedure [20]. Medial edge of the orbit was dissected free and orbital contents were gently elevated using small cotton balls. The infraorbital nerve was dissected and two silk ligatures (5-0) were placed around the nerve spaced 2 mm apart. The ligatures reduced the diameter of the nerve by just a noticeable amount and they did not interrupt the epineural circulation, as previously described [21]. Orbital content was then gently moved back to orbit. Skin and underlying fascia were sutured in layers with nylon sutures (4-0). Sham operation was performed by exposing infraorbital nerve without placing silk ligatures.

### Mechanical allodynia testing

Von Frey monofilaments (Stoelting Co., Wood Dale, IL, USA) were used for mechanical stimulation. The filaments produced a bending force of 0.16, 0.4, 0.6, 1, 2, 4, 6, 8, 10 and 15 g. Testing was performed as previously described in detail by Kayser et al. [17]. The rats were placed in a small transparent plastic cage for ten minutes to accommodate to the experimental environment until they assumed their normal sniffing/no locomotion position. For each session, series of von Frey filaments were applied on the tested side of the face in ascending order, starting at 0.16 g, until a defined behavioral response was elicited. Each time, the measurement started on the side contralateral to IoNC injury. A positive reaction to the stimulation within the IoN territory, at the center of the vibrissal pad area, consisted of a rapid withdrawal of the head and/or attack/escape reaction [15]. If no response was observed, we assigned 15 g as the withdrawal threshold, since the pressure exerted by thickest (15 g) filament was enough to push the head of sham-operated rats. Measurements were performed three times for each filament on both sides of the face in 10 min intervals. Baseline measurements were performed one day prior to the IoNC injury. Pain sensitivity was retested fourteen days following the IoNC injury (day 0). Time between the surgery and testing was chosen based on the time required for full development of mechanical sensitivity in IoNC model [15], [17] and aimed to exclude the possible influence of postsurgical hyperalgesia on pain testing [9]. Only animals which developed bilateral mechanical allodynia (we defined it as responsiveness to 0.16–2 g on both sides) were included in the further study (approximately 70% of the IoNC operated animals).

### Dural extravasation

Evans blue (dye which complexes with plasma proteins) technique is routinely used to investigate the effects of nociceptive afferents on vascular function [3], [22]. Animals were injected with 1 ml Evans blue solution (40 mg/kg) into the tail vein. After stimulation of the vibrissal pad area with von Frey filament (2 g) for a period of 10 minutes, animals were deeply anesthetized by chloral hydrate (300 mg/kg, i.p), the thorax was opened and the right atrium incised for drainage. Saline (500 ml) was perfused via left ventricle at a constant rate. The brain was carefully removed and the cranial cavity rinsed with saline to remove residual blood and cerebrospinal fluid prior to dissection of the dura. The dura covering supratentorial region of the brain, primarily innervated by trigeminal nerve [23], was harvested from left and right side separately. To assess bilateral dural extravasation, dural tissue from 4 animals was pooled in one sample (left and right side separately) and weighted. Average weight was 10–18 mg. Collected tissue was incubated in 2 ml of formamide at 37°C for 48 h. Colorimetric absorbance measurements of Evans blue formamide extracts were carried out with a spectrophotometer (Iskra, Ljubljana, Slovenia) at 620 nm.

### Experimental design

#### Time course of bilateral mechanical allodynia

Animals which developed mechanical allodynia on day 14 after IoNC were divided in 4 groups (5–8 animals): 1. Sham operated; 2. IoNC+Saline; 3. IoNC+BoNT/A (ipsi.) (BoNT/A injected ipsilateral to the IoNC injury); 4. IoNC+BoNT/A (contra.) (BoNT/A injected contralateral to the IoNC injury). Measurements of mechanical sensitivity were further repeated on day 3, 6, 20 and 30 following BoNT/A single injection (day 0).

#### Bilateral assessment of dural neurogenic inflammation

Plasma dural extravasation was investigated in additional 4 groups of animals (20 animals per group): 1. Sham operated; 2. IoNC+Saline; 3. IoNC+BoNT/A (ipsi.); 4. IoNC+BoNT/A (contra.) Fourteen days after the IoNC injury, neuropathic animals were selected similarly to that described above, and injected with BoNT/A or saline. Three days after the injection, allodynia was retested and Evans blue technique was employed to test dural plasma protein extravasation.

#### Characterization of axonal transport of BoNT/A

To test the role of axonal transport of BoNT/A in sensory neurons for its effects on bilateral neuropathic pain and dural extravasation, an axonal transport blocker colchicine was injected into the trigeminal ganglion.

After 24 hrs, animals pre-treated with colchicine were injected s.c. into the vibrissal pad with BoNT/A or saline into the side ipsilateral to the IoNC injury and colchicine injection.

Animals were divided in 5 groups: Sham; IoNC+saline (i.g.)+saline (s.c.): IoNC+saline (i.g.)+BoNT/A (s.c.); IoNC+colchicine (i.g.)+saline (s.c. ); IoNC+colchicine (i.g)+BoNT/A (s.c.).

Experimental procedure for bilateral measurements of mechanical allodynia and dural extravasation was similar to that described above, except that the dural samples were not divided into left and right sides and were not pooled, because we observed no significant difference between dural extravasation on opposite sides.

#### Orofacial formalin test

Animals pretreated with saline or BoNT/A (3.5 U/kg into the vibrissal pad) 3 days prior to testing were injected with Evans blue. Immediately following Evans Blue administration the animals were injected unilaterally with 2.5% formalin (50 µl) into the BoNT/A treated vibrissal pad and placed in transparent cages for 45 min of observation. Behavioral testing was performed as previously described [18], [24]. In brief, facial rubbing time (in seconds) was measured in 3 minute intervals for a period of 45 min.

Following behavioral testing, animals were immediately anesthetized and perfused with saline. Left and right sides of dural tissue were excised and pooled from two animals.

Animals were divided in 3 groups (8 animals per group): 1. control: saline (s.c.); 2. saline (s.c.)+formalin (s.c.); 3. BoNT/A (s.c.)+formalin (s.c.).

### Statistical analysis

The results were presented as mean ± S.E.M and analyzed by ANOVA followed by the Newman-Keuls post hoc test for between-group differences. In the time-course experiment ANOVA was employed for repeated measurements followed by Tukey's test. P<0.05 was considered significant.

## Results

### Effects of BoNT/A on allodynia induced by IoNC injury

**Figure 1:**
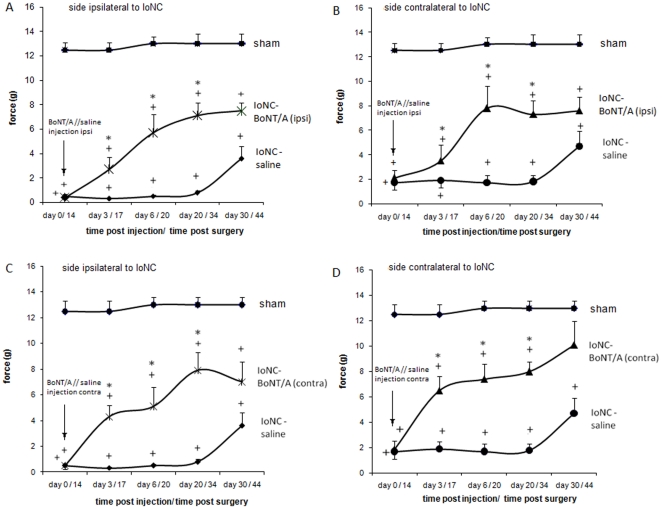
Botulinum toxin A (3.5 U/kg) decreases mechanical allodynia induced by IoNC- injury in rats. BoNT/A was injected into the vibrissal pad ipsilaterally (A and B) or contralaterally (C and D) to the side of the nerve injury. Measurements were performed on the ipsilateral (A and C) and contralateral (B and D) side to the nerve injury. Legend: ▪ – sham-operated control; ♦ - saline-injected IoNC operated animals when measured on the ipsilateral side; • - saline-injected IoNC operated animals when measured on the contralateral side; 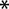 - BoNT/A-injected IoNC operated animals when measured on the ipsilateral side; ▴ – BoNT/A-injected IoNC operated animals when measured on the contralateral side. Mean ± S.E.M; n = 5–8 (n- indicates the number of animals per group). *P<0.01 - BoNT/A-injected compared to saline-injected animals; ^+^P<0.001 - BoNT/A-injected or saline-injected animals compared to sham-operated control.

BoNT/A (3.5 U/kg) injected s.c. into the vibrissal pad (ipsilaterally to the nerve injury) of animals which developed bilateral allodynia 14 days after the IoNC significantly reduced mechanical allodynia on that side, as well as on the contralateral side (Figure 1A and 1B). When BoNT/A was injected contralaterally to the nerve injury, it still reduced mechanical allodynia on both sides of the face (Figure 1C and 1D). The effect on both sides was evident on our first measurement 3 days post BoNT/A injection, and persisted for at least 17 more days. On day 44 following the IoNC injury (day 30 post-BoNT/A application), mechanical allodynia started to disappear (Figure 1 A, B, C, D). To exclude the possibility of BoNT/A passive diffusion away from the site of injection, methylene blue (20 µl) was injected into the vibrissal pad. The colour only resided near the place of injection and did not spread away from the site of injection (data not shown). Moreover, animals injected into the vibrissal pad with 3.5 U/kg of BoNT/A or even higher doses (15 U/kg) did not exhibit impaired rotarod performance which excludes possible systemic effect (results not shown).

### Effects of BoNT/A on dural extravasation after IoNC injury

**Figure 2:**
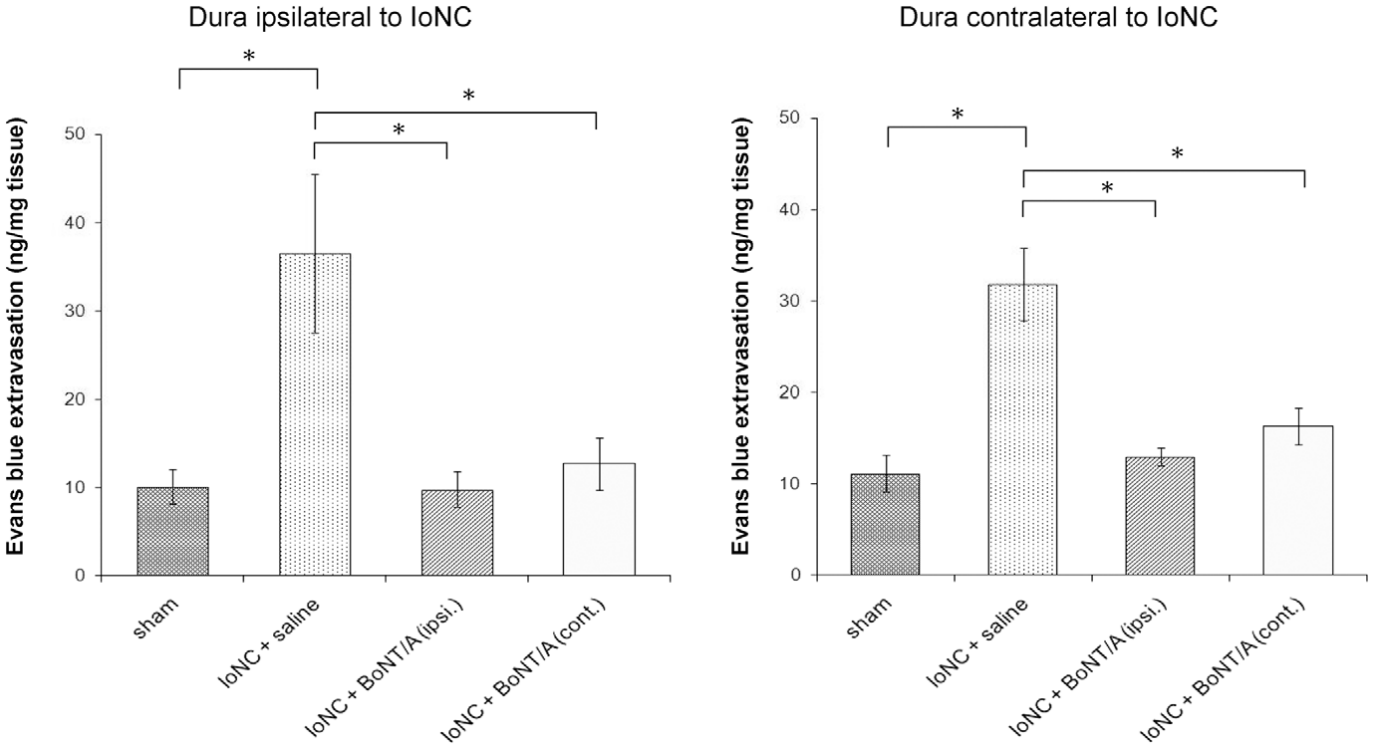
Botulinum toxin A (3.5 U/kg) decreases dural extravasation in IoNC injured rats. BoNT/A was injected into the vibrissal pad 14 days after infraorbital constriction ipsilaterally or contralaterally to the side of nerve injury. Absorbances of Evans blue extracted from dural tissue after incubation in formamide were measured. Each sample consists of combined dural tissue of 4 animals. Corresponding amounts of Evans blue were calculated from calibration curve, and divided by the wet mass of corresponding dural samples. Legend: Sham-operated animals; *IoNC+saline* – saline injected animals after IoNC injury; *IoNC+BoNT/A ( ipsi.)* – BoNT/A injected ipsilaterally in IoNC operated animals; *IoNC+BoNT/A (contra.)* – BoNT/A injected contralaterally in IoNC operated animals. Mean ± S.E.M., n = 5 (n-indicates the number of samples per group). *P<0.01 (Newman Keuls post-hoc).

In the IoNC operated saline treated rats with bilateral mechanical allodynia, dural extravasation (measured as ng of Evans blue per mg of dural tissue) was measured three days after BoNT/A injection into the vibrissal pad, i.e. 17-days post-IoNC injury. BoNT/A (3.5 U/kg) abolished bilateral dural extravasation in the IoNC injured rats (Figure 2). Botulinum toxin exerted the effect on dural extravasation when injected both ipsilaterally or contralaterally to the site of nerve injury (Figure 2).

### Effect of BoNT/A on mechanical allodynia and dural extravasation after IoNC injury is axonal transport dependent

**Figure 3:**
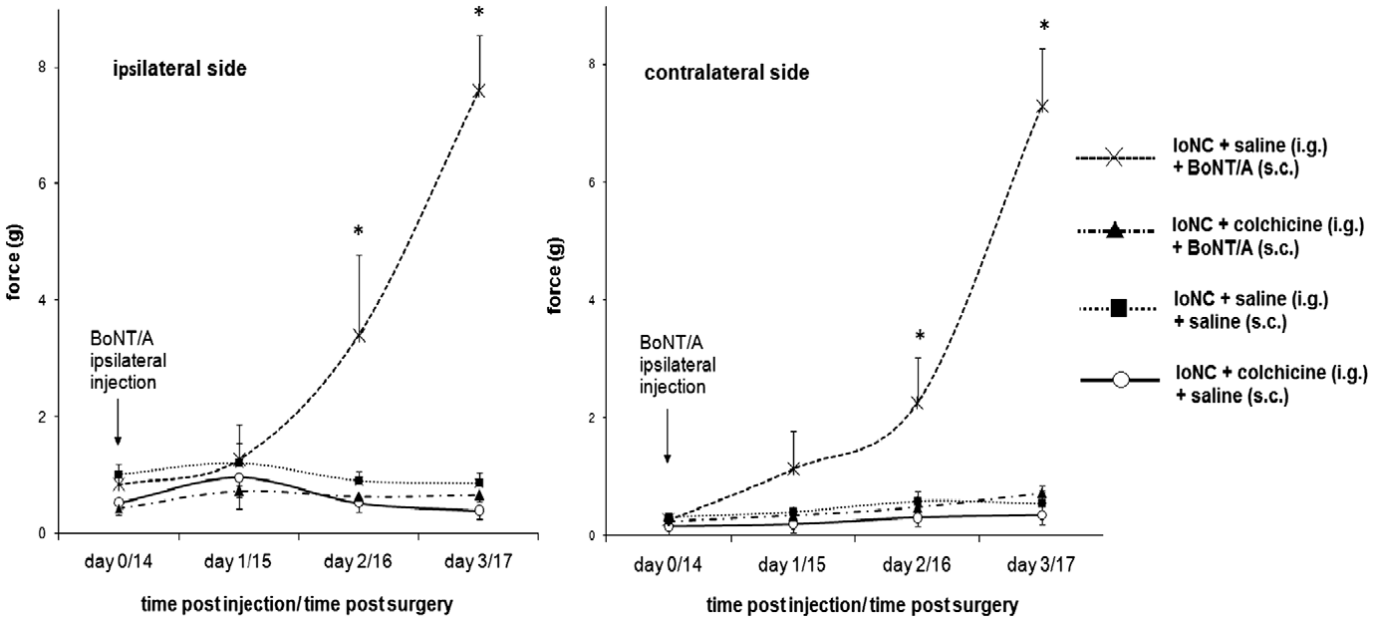
BoNT/A (3.5 U/kg) decreases IoNC-induced mechanical allodynia which is prevented by intraganglionic colchicine pretreatment. Animals were injected into the trigeminal ganglion with colchicine 24 hrs prior to s.c. BoNT/A or saline injection into the vibrissal pad (both injections given into the ipsilateral side to the IoN injury). Animals were divided in 5 groups: Sham operated; IoNC+saline (i.g.)+saline (s.c.); IoNC+saline (i.g.)+BoNT/A; IoNC+colchicine (i.g.)+saline (s.c.); IoNC+colchicine (i.g.)+BoNT/A (s.c.). Measurements were performed on the ipsilateral and contralateral side to the nerve injury. Sham operated values (around 12.5±0.5 g) were not shown. Mean ± S.E.M., n = 6 (n-indicates the number of animals per group). *P<0.01 compared to IoNC+saline (i.g.)+saline (s.c.); IoNC+colchicine (i.g.)+saline (s.c.); IoNC+colchicine (i.g.)+BoNT/A (s.c.) (Tukey's test).

**Figure 4:**
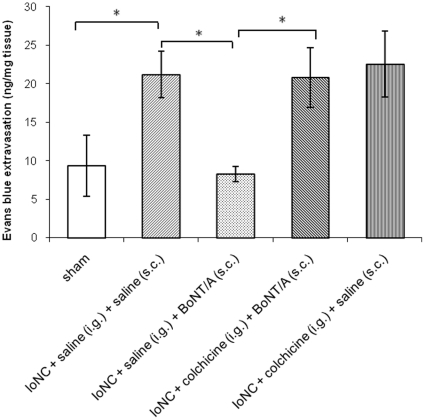
BoNT/A (3.5 U/kg) decreases dural extravasation in IoNC-injured rats which is prevented by intraganglionic colchicine. Animals were injected into the trigeminal ganglion with colchicine 24 hrs prior to s.c. BoNT/A or saline injection into the vibrissal pad (both injections given into the ipsilateral side to the IoN injury). Absorbances of Evans blue extracted from dural tissue after incubation in formamide were measured. Legend: i.g. - intraganglionic; s.c. – subcutaneous injection. Data are represented as mean ± S.E.M., n = 6 (n-indicates the number of samples per group). *P<0.01 (Newman Keuls post-hoc).

Mechanical bilateral allodynia and dural extravasation were significantly reduced 2 and 3 days following the BoNT/A injection into the vibrissal pad. When axonal transport blocker colchicine was injected ipsilateral to BoNT/A injection into the trigeminal ganglion, BoNT/A failed to reduce bilateral mechanical allodynia (Figure 3) and dural neurogenic extravasation (Figure 4). Colchicine itself did not alter either mechanical allodynia (Figure 3) or dural neurogenic inflammation induced by IoNC (Figure 4), although it was previously shown that it affects thermal hyperalgesia in experimental animals [25].

### Orofacial formalin test

BoNT/A (3.5 U/kg) reduced formalin-induced painful behaviour, measured as time of facial rubbing, during phase II of formalin test (results not shown), which is in line with our previous findings [18].

**Figure 5:**
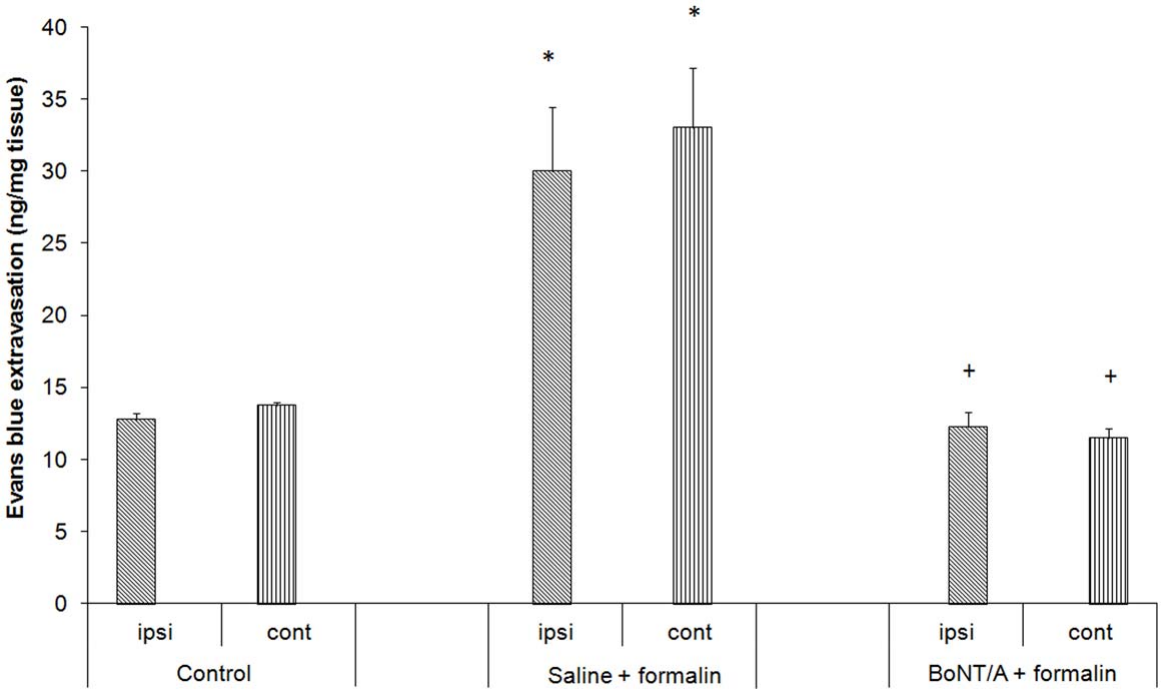
BoNT/A (3.5 U/kg) decreases orofacial formalin-induced dural extravasation. Dural extravasation was induced by formalin injection into the vibrissal pad (2.5% formalin, 50 µl). Ipsilateral vibrissal pad was injected with saline of BoNT/A 3 days prior to formalin injection. Amount of Evans blue extracted from dural tissue after incubation in formamide was measured. Ipsi: dural tissue ipsilateral to injections; Cont: dural tissue contralateral to injections. Control: saline -injected animals; saline+formalin: animals injected with saline and formalin; BoNT/A+formalin: animals injected with BoNT/A and formalin. Data are represented as mean ±SEM. n = 4 (n –number of samples/group), *,- P<0.05 compared to control; + - P<0.05 compared to saline+formalin (Newman-Keuls post hoc).

Formalin-induced orofacial pain also resulted in bilateral dural extravasation (measured as amount of Evans-blue extravasated dye), similarly to IoNC. Unilaterally applied BoNT/A reduced dural extravasation on both sides, as well (Figure 5).

## Discussion

### Trigeminal pain-evoked dural protein extravasation

Dural neurogenic inflammation is a phenomenon which is only superficially investigated. There is sparse evidence for its existence in humans [26] and it was primarily investigated in relation to migraine in an animanl model [27]–[31]. According to trigeminovascular theory, migraine is associated with activation of trigeminal nerve afferents which innervate dura, leading to release of neuropeptides like calcitonin gene related peptide (CGRP) and substance P (SP) [32]. It can be evoked by electrical stimulation of trigeminal ganglion and systemic chemical or immunological stimulation [33]. Classical antimigraine agents (like ergot alkaloids, triptans), decrease dural plasma protein extravasation after electrical stimulation of trigeminal ganglion [27]–[31].

As a new finding of this study we have shown that dural extravasation can be induced by two different types of trigeminal pain (neuropathic and inflammatory).

Nerves which innervate the dura (middle meningeal nerve) and area innervated by infraorbital nerve (affected by IoNC and formalin) belong to the maxillary trigeminal branch, but they are distinct and physically separated [23]. Possible explanations for dural extravasation after IoNC and formalin could be: (1) interactions between neurons innervating the injured infraorbital nerve area with adjacent neurons in trigeminal ganglion innervating dural vessels, and/or (2) central cross-excitation inside spinal trigeminal nucleus in the brainstem. Mutual activation between the adjacent neurons of the same ganglion has been described in trigeminal and dorsal root ganglia [34], [35]. Based on the animal model of chemical stimulation of dura, it was proposed that the coexistence of dural extravasation and allodynia is modulated by excitation of nociceptive neurons in trigeminal nucleus caudalis, a convergent point of sensory inputs from dura and facial skin [36], [37].

Allodynia and dural extravasation on the side contralateral to nerve injury might be associated with spreading of excitation to contralateral nuclei, via unknown neural pathways. It is shown that unilateral electrical stimulation of trigeminal ganglion results in bilateral decrease of neuropeptides, suggesting the activation of contralateral trigeminal pathway [38]. Using unilateral injection of retrograde tracers, Jacquin et al. [39] found that some primary trigeminal afferents cross the midline and terminate in contralateral dorsal horn, which might explain our observation.

### Bilateral effect of BoNT/A on allodynia and dural extravasation

It was recently demonstrated that BoNT/A counteracts the development of unilateral neuropathy which follows IoNC [7], [8]. Previously mentioned studies used a different experimental setup: contralateral side was sham-operated, and BoNT/A was administered before the development of neuropathy. In our present study, sham operation was done on a separate group of animals and, additionally, we administered BoNT/A following the full development of neuropathic pain. Using this approach, we have observed BoNT/A bilateral effects on allodynia, lasting over a period of one month. This effect was seen even when BoNT/A was applied to the uninjured side (Figure 1).

Bilateral pain reduction after unilateral peripheral BoNT/A injection has recently been demonstrated in models of paclitaxel-induced polyneuropathy [14], diabetic neuropathy [13] and acidic saline-induced mechanical hyperalgesia in rats [12]. Bach-Rojecky and Lacković [12] demonstrated that BoNT/A effects are CNS mediated and dependent on axonal transport. Apart from bilateral pain reduction in IoNC, effect of BoNT/A was bilateral in both IoNC and formalin-evoked dural extravasation. Contralateral effect of BoNT/A might ensue via contralaterally terminating afferent sensory fibers [39] or by transcytosis within central neurons, as suggested by some authors [7], [40].

The mechanism of BoNT/A action on pain and dural extravasation is unclear. It might be connected with BoNT/A effects on CGRP/SP release. In several *in vitro* studies on cultured trigeminal ganglion neurons or brainstem slices, BoNT/A reduced stimulated CGRP release [41]–[43]. In addition, BoNT/A reduces Substance P release in rabbit iris sphincter [44]. Since an increased level of CGRP might be important in vasodilatation and transmission of pain in migraine, some authors propose that BoNT/A effectiveness in migraine is associated with reduced CGRP release from trigeminal neurons [41], [45], [46].

### Axonal transport of BoNT/A in sensory trigeminal neurons

Axonal transport of BoNT/A has recently been demonstrated in motoneurons [40] and sensory neurons [18]. Truncated SNAP-25 was immunohistochemically demonstrated in medullary dorsal horn after BoNT/A (3.5 U/kg) injection into the rat vibrissal pad [18]. Apparently, BoNT/A enters peripheral trigeminal nerve endings and is axonally transported through the trigeminal ganglion to the spinal trigeminal nucleus.

Since axonal transport to CNS was shown to be necessary for antinociceptive activity of BoNT/A [12], [18], we hypothesized that bilateral reduction of allodynia and dural extravasation are dependent on BoNT/A axonal transport in sensory neurons. In line with this hypothesis is our present finding that BoNT/A effectively reduced pain and dural extravasation even when applied to uninjured side of neuropathic animals.

Colchicine, prevented both effects of peripherally applied BoNT/A, most probably by blocking axonal transport of BoNT/A in trigeminal sensory neurons. These results additionally exclude bilateral effect due to possible peripheral spreading to uninjected side of the face. Importantly, this experiment also shows that trigeminal sensory system is the primary site of BoNT/A action and excludes possible involvement of extracranial autonomous nerves.

### Conclusion

Present results demonstrate for the first time that bilateral dural neurogenic inflammation can be evoked by experimental pain in trigeminal region. Both pain and dural neurogenic inflammation can be prevented by single BoNT/A peripheral injection, provided by axonal transport in sensory neurons. Although the central site of BoNT/A action seems the only logical explanation, the precise mechanism requires further elucidation. The possibility that dural extravasation occurs in other types of extracranial pain and its importance for trigeminal pain pathophysiology should be further investigated.
